# Moonlighting Proteins: Diverse Functions Found in Fungi

**DOI:** 10.3390/jof9111107

**Published:** 2023-11-15

**Authors:** Nicole J. Curtis, Krupa J. Patel, Amina Rizwan, Constance J. Jeffery

**Affiliations:** 1 Department of Biological Sciences, University of Illinois at Chicago, Chicago, IL 60607, USA; ncurtis2@uic.edu (N.J.C.); kpate353@uic.edu (K.J.P.); 2 Bay Path University, Longmeadow, MA 01106, USA; arizwan@baypath.edu

**Keywords:** moonlighting protein, multifunctional, RNA binding proteins, multiprotein complex, enzymes, transcription factor

## Abstract

Moonlighting proteins combine multiple functions in one polypeptide chain. An increasing number of moonlighting proteins are being found in diverse fungal taxa that vary in morphology, life cycle, and ecological niche. In this mini-review we discuss examples of moonlighting proteins in fungi that illustrate their roles in transcription and DNA metabolism, translation and RNA metabolism, protein folding, and regulation of protein function, and their interaction with other cell types and host proteins.

## 1. Introduction

In the million years since fungi split from other eukaryotes, they evolved a wide diversity of taxa with a variety of morphologies and sometimes complex life cycles, adapted to numerous ecological niches. They also developed diverse mechanisms of nutrient acquisition, including secreting digestive enzymes, symbiosis, or even parasitism. All these developments required the evolution of new protein functions. Moonlighting proteins have arisen throughout the evolutionary tree as a means of enabling a vast increase in protein functions, as well as a mechanism to coordinate many functions. A moonlighting protein is a single polypeptide chain with two or more physiologically relevant biochemical or biophysical functions [1]. Moonlighting proteins can have a second function by interacting with another protein, DNA, or RNA, or with different ligands or cofactors; by joining a multiprotein complex; or by being expressed in a different cell type. They can also have different functions at different times in development or stages of differentiation; in different locations within the cell, for example, in the cytoplasm or nucleus; or when secreted or bound to the cell surface.

As shown in the examples below and in Table 1, moonlighting proteins have been found in a variety of fungal taxa [2]. Not surprisingly, additional functions were often first found in model organisms that are used in genetic or biochemical studies including *Saccharomyces cerevisiae* (budding yeast), *Neurospora crassa* (filamentus fungi, mold), *Schizosaccharomyces pombe* (fission yeast), and *Emericella nidulans* (*Aspergillus nidulans*). A few examples were identified in *Komagataella pastoris* (formerly known as *Pichia pastoris*), a budding and spore-forming yeast that is widely used as a model organism and also as an expression system for protein production in research and biotech industries. Other moonlighting proteins were found in studies of fungi that play roles in health and disease or are used in industry or food production—*Paracoccidioides brasiliensis* is a dimorphic fungus that can cause paracoccidioidomycosis, *Histoplasma capsulatum* causes histoplasmosis, *Cryptococcus neoformans* can cause a pneumonia-like disease or meningitis in people with weakened immune systems, and *Sporothrix schenckii* causes sporotrichosis, a subcutaneous mycosis. In addition, several *Candida* species are a normal part of human gut flora, but can also act as opportunistic pathogens. *Kluyveromyces lactis* (formerly *Saccharomyces lactis*) is used in genetic research and also in industry for production of chymosin (rennet) for cheese production, and *Tuber melanosporum* has fruiting bodies that are eaten as truffles.

**Figure 1:**
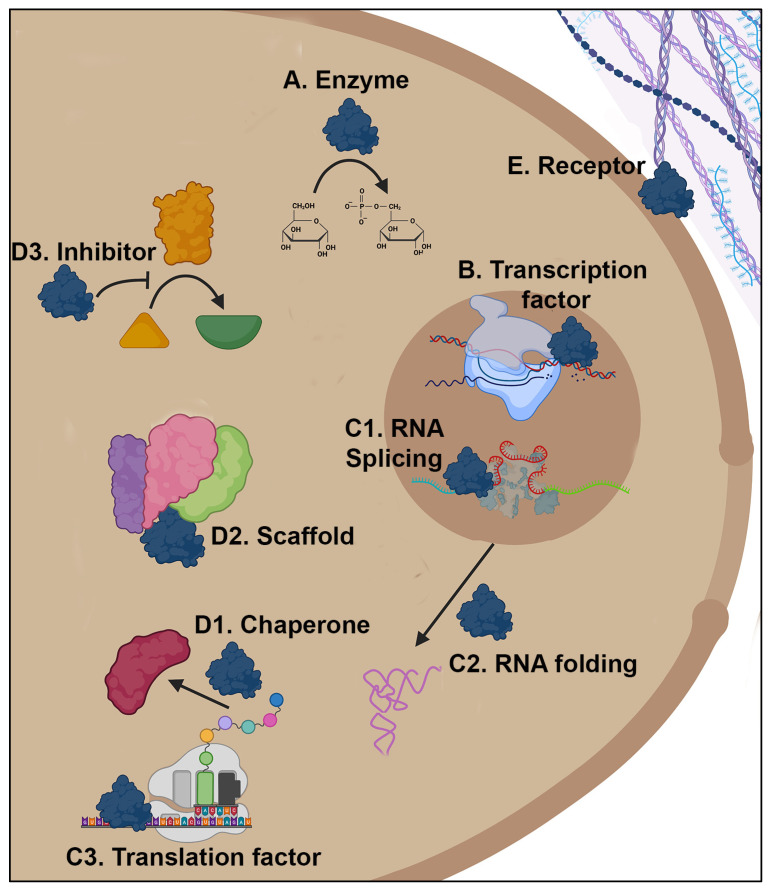
Examples of multiple functions of fungal moonlighting proteins. A moonlighting protein (dark blue) can have a function as an enzyme in the cytoplasm (A). It could have an additional function as a DNA binding transcription factor in the nucleus (brown circle, B). By binding to RNA the moonlighting protein could affect splicing (C1), folding of the RNA (magenta, C2), and/or translation by the ribosome (C3). It could interact with another protein (red) to act as a chaperone to aid in protein folding (D1), or act as a scaffold that brings multiple proteins (green, pink, purple) together in a metabolon or as a subunit of a multiprotein complex (D2), or it could inhibit the function of another protein (yellow, D3). An intracellular protein could have an additional function on the cell surface where it interacts with host proteins, such as components of the extracellular matrix (purple and blue, E). Created with BioRender.com.

In this mini-review we discuss examples of moonlighting fungal proteins. Most of the proteins are either enzymes or chaperones with at least one additional function. For clarity, we are grouping the proteins into four groups based on whether the additional function plays a role in 1. affecting transcription, DNA metabolism, or DNA maintenance; 2. affecting translation or RNA metabolism; 3. affecting another protein’s function; or 4. interacting with host cells or proteins (Figure 1). Additional fungal moonlighting proteins are described in other papers in this collection, including surface proteases that have additional functions such as acting as adhesins to host proteins [72]; cell surface adhesins with additional functions [73]; and a protein with different functions in multiple protein complexes, Hal3 [38].

## 2. Examples of Moonlighting Proteins in Fungi

### 2.1. Affecting Transcription, DNA Metabolism, or DNA Maintenance

A variety of fungal enzymes have been found to have a second function in regulating transcription or in DNA metabolism (Table 1). Enzymes in carbohydrate, nitrogen, and sulfur metabolism, as well as kinases and chaperones, have been found to regulate transcription either by binding directly to DNA or by binding to and regulating transcription factors. *S. cerevisiae* hexokinase (Hxk2) and a homologue in *K. lactis*, galactokinase, phosphorylate hexoses in glycolysis and other pathways in carbohydrate metabolism. High cellular glucose concentrations cause Hxk2 to move to the nucleus where it interacts directly with Mig1, which binds to promoters of genes that are repressed by glucose [13]. Galactokinase is a transcriptional activator of GAL genes [5,6].

Examples in nitrogen metabolism include *S. cerevisiae* Arg5,6 and Ure2. Arg5,6 (N-acetyl-gamma phosphate reductase and acetyl glutamate kinase) in the arginine biosynthetic pathway binds to mitochondrial and nuclear DNA *in vivo* and acts as a regulator of transcription for several genes [10]. Ure2 has glutathione peroxidase activity and also functions in nitrogen catabolite repression by binding to transcriptional activators Gln3 and Gat1. Binding by Ure2 keeps them in the cytoplasm and prevents transcription of their target genes [19,20,21,22].

An example in sulfur metabolism was found in the Perigord black truffle, *T. melanosporum* (a filamentous mycorrhizal ascomycetous fungus). Phosphoadenosine phosphosulfate reductase is a thioredoxin that functions in sulfur assimilation, by converting activated sulfate (PAPS) to sulfite (adenosine 3′,5′-bisphosphate + sulfite + thioredoxin disulfide <-> 3′-phosphoadenylyl sulfate + thioredoxin). It also has an additional function as a transcription factor [8].

Several other types of enzymes or chaperones help the cell respond to changes in the environment by regulating the actions of transcription factors. *S. cerevisiae* Arg82 kinase phosphorylates inositol 1,4,5-trisphosphate (Ins(1,4,5)P3) and inositol 1,3,4,5-tetrakisphosphate (Ins(1,3,4,5)P4) and has a second function in stabilizing the transcription factors Arg80 and Mcm1 [11]. *S. cerevisiae* zuotin, a component of the ribosome-associated chaperone complex (RAC) that helps in folding of nascent polypeptide chains, is also an activator of Pdr1 transcription factor [23]. Superoxide dismutase breaks down superoxide (O_2_^−^) to hydrogen peroxide (H_2_O_2_), and it also acts as a transcription factor. In response to H_2_O_2_, it regulates expression of genes involved in oxidative resistance and DNA damage repair. Treatment with reactive oxygen species (ROS) increases Sod1 binding to the promoters of *rnr3* and *gre2* [17,18]. *S. cerevisiae* glyceraldehyde 3-phosphate dehydrogenase (GAPDH), affects gene expression more generally. It binds to Sir2 and regulates gene silencing by influencing Sir*2’*s association with chromatin, especially near telomeres [12]. *T. melanosporum* Ask1 protein is part of the DASH complex, which binds to microtubules and kinetochores and regulates their association. It also interacts with DNA in a separate function as a transcriptional regulator [7].

Other enzymes and chaperones have additional functions in DNA damage repair and maintaining mitochondrial DNA. Lys20 homocitrate synthase in the lysine biosynthetic pathway assists in Ino80 accumulation at DNA breaks to aid in histone eviction for DNA damage repair [16]. *S. cerevisiae* Hsp60 chaperonin is a protein folding chaperone that has a second function in which it binds the template strand of active mtDNA ori sequences and is involved in the structure and transmission of mitochondrial DNA nucleoids to daughter cells [15]. *S. cerevisiae* acetohydroxyacid reductoisomerase, an enzyme in L-leucine, L-isoleucine, and L-valine amino acid biosynthesis, is also involved in maintaining mitochondrial DNA stability enzymes [9].

Moonlighting proteins can also be involved in both DNA and RNA metabolism. *E. nidulans* (*A. nidulans*) I-AniI is an mRNA maturase that is required for splicing of the intron in the cytochrome b (*cobA*) gene, which contains the coding sequence for the maturase. I-Anil stimulates the intrinsic ribozyme activity of the intron by binding to and stabilizing the three-dimensional structure of the RNA. I-Anil is also a homing endonuclease that introduces a double-strand break at a specific location in the c*obA* gene and helps in the insertion of a group I intron containing its own coding sequence [3,4].

### 2.2. Affecting Translation or RNA Metabolism

In many branches of the evolutionary tree, enzymes from intermediary metabolism, amino acid tRNA synthetases, and protein components of the ribosome have been found to have a second function in RNA metabolism, including splicing, folding, regulation of translation, cellular localization, or RNA lifetime (Table 1).

In *S. cerevisiae*, several protein components of the ribosome interact with RNA as a second function. The S14 protein of the small subunit (40S) binds to an RNA stem-loop and represses expression of *rps14B* pre-mRNA [29]. The L32 protein of the large subunit (60S) inhibits the splicing of the transcript of its own gene, *rpl32* [27]. The L2 protein of the large subunit shortens the half-life of L2 mRNA [26]. The S28 component of the small subunit also shortens the half-life of its own mRNA by binding to a hairpin structure in 3′ UTR and also to the decapping machinery [30].

*S. cerevisiae* isocitrate dehydrogenase is an enzyme in the citric acid cycle. Both subunits 1 and 2 were found to bind to 5′-untranslated leaders of mitochondrial mRNAs [25]. *S. cerevisiae* leucyl-tRNA synthetase, which attaches L-leucine to tRNA(Leu) for use in protein synthesis, is involved in group I intron splicing [28]. The *N. crassa* tyrosyl tRNA synthetase promotes folding of the group I intron catalytic core [24].

The number of examples of enzymes with an additional function in RNA metabolism is likely to grow significantly. In recent years, high-throughput proteomics studies have identified many other fungal proteins that bind RNA [74,75,76,77,78,79,80]. For example, *S. cerevisiae* RNA-binding proteins are summarized in the RBP2Go database [81], and they include all of the enzymes in glycolysis and the citric acid cycle. For many of the enzymes, it is not known if RNA binding is indicative of a second function in translation, RNA stability, localization, or splicing, or another RNA function, or if the RNA binding could, instead, be involved in regulation of the enzyme function. More experiments are needed to clarify the role and mechanisms of interactions with RNA.

### 2.3. Affecting Another Protein’s Activity

In addition to regulating protein function through regulation of transcription or translation, the actions of the many biochemical pathways and processes within a cell are coordinated through protein–protein interactions. In some cases, moonlighting proteins with a catalytic function have a second function where they interact with other proteins to facilitate biogenesis, folding, or assembly of other proteins or protein complexes (Table 1). *S. cerevisiae* arginase catalyzes the reaction L-arginine + H_2_O => L-ornithine + urea for breakdown and the use of arginine as a nitrogen source. Arginase can also regulate arginine biosynthesis. It senses changes in cellular ornithine and arginine concentrations and responds by binding to and inactivating the first enzyme in arginine biosynthesis, ornithine transcarbamylase [34].

*S. cerevisiae* peroxiredoxins 1 and 2 have a peroxidase antioxidant function. In response to changing cellular redox conditions, they switch to act as general molecular chaperones that help proteins fold [40]. Other moonlighting enzymes function in the folding or assembly of specific proteins or complexes. *Hansenula poymorpha* (*Pichia angusta*) pyruvate carboxylase, an enzyme in gluconeogenesis, has a second function in the assembly of peroxisomal alcohol oxidase (AOX). It may mediate FAD binding to the AOX monomers in the cytoplasm, which enables them to be transported to the peroxisome where they can assemble into the active octameric form [31,32]. *S. cerevisiae* fructose 1,6-bisphosphate aldolase, an enzyme in glycolysis and gluconeogenesis, has been found to bind to the vacuolar H+-ATPase and is needed for its assembly. The aldolase catalytic activity is not needed for this function [37]. *S. cerevisiae* subunit 3 of succinate dehydrogenase is a part of respiratory complex II in the electron transport chain in mitochondria, and it also helps in the biogenesis and assembly of membrane-integral subunits of the TIM22 complex, a mitochondrial inner membrane translocase [43,44,45,46].

Another way proteins can affect other protein functions is through acting as a scaffold to bring proteins or enzymes together. The *S. cerevisiae* RACK1 component of the 40S (small) ribosomal subunit acts as a scaffold in cytoplasmic signal transduction pathways [41,42].

Proteins can also help the cell respond to changes in the environment by switching from one function to serving as a signaling molecule to promote a different cellular process. *S. cerevisiae* cytochrome c is an electron carrier protein component of the mitochondrial electron transport chain. When released from the mitochondria it has a second function of binding to apoptosis protease activation factor-1 (Apaf-1) to promote apoptosis [35]. *P. pastoris* phosphofructokinase, an enzyme in glycolysis, is required for microautophagy (vacuolar degradation of peroxisomes). The catalytic activity is not needed, and only the alpha subunit of phosphofructokinase plays this role [33]. *S. cerevisiae* enolases 1 and 2 are required for vacuole homotypic membrane fusion and protein trafficking to the vacuole, and their catalytic activity is not needed for this role [36].

Yeast (*S. pombe*, *S. cerevisiae*) Hal3 proteins are found in two different heteromultimeric enzymes. As part of phosphopantothenoylcysteine decarboxylase, Hal3 helps catalyze the decarboxylation of phosphopahtothenoyl L-cysteine in coenzyme A biosynthesis. It also acts as a regulatory subunit of a serine/threonine phosphatase, Pzh1 or Ppz1 [39,82]. The multiple functions of Hal3 are described in more detail in another paper in this collection by Casamayor and Arino [38].

### 2.4. Intracellular Proteins with an Additional Function on the Cell Surface

Intracellular proteins that have a second function when outside the cell and bound to the cell surface comprise another type of moonlighting protein found throughout the evolutionary tree [83,84,85]. Both commensal and pathogenic species use these proteins as adhesins to interact with host cells and proteins: for example, fibronectin, laminin, and other proteins in the extracellular matrix (Table 1). Microbial pathogens often employ moonlighting proteins to bind to host plasminogen, helping convert it to the active protease plasmin so that the protease can aid in invading host tissues [86,87,88]. In some cases, pathogens use moonlighting surface proteins to bind directly to proteins of the immune system and interfere with their activity. In this section we summarize examples of intracellular proteins moonlighting on the cell surface in several species. For additional examples and information, Arvizu-Rubio and coworkers have recently published a more extensive list of moonlighting proteins on the surface of pathogenic fungi [89].

Due to roles as both commensal organisms and as opportunistic pathogens, several groups have identified moonlighting proteins on the *Candida* cell surface, including enzymes in carbohydrate metabolism and protein synthesis, chaperones, and enzymes in redox homeostasis (reviewed in [90]). *C. albicans* glyceraldehyde 3-phosphate dehydrogenase (GAPDH) binds to fibronectin and laminin in the extracellular matrix [53]. Triosephosphate isomerase from *C. albicans* and *C. glabrata* binds to several extracellular matrix proteins [61]. Enolase from *C. albicans, C. tropicalis, and S. cerevisiae* binds to vitronectin, fibronectin, and plasminogen [63]. *C. albicans* glutathione reductase [54,55] and phosphotransferase [48,62] bind to plasma proteins. *C. tropicalis* fructose-bisphosphate aldolase and enolase 1 from glycolysis [64] bind fibronectin, vitronectin, and laminin. *C. albicans* heat shock protein Ssa2, a chaperone, binds to HTN3/histatin 5, a peptide from human saliva [57]. *H. capsulatum* Hsp60 chaperone [66], *P. brasiliensis* triose phosphate isomerase (glycolysis and gluconeogenesis) [69], and *Talaromyces marneffei* (*Penicillium marneffei)* GAPDH [71] were all found to act as adhesins. *P. brasiliensis* enolase (glycolysis and gluconeogenesis) binds fibronectin [67], and *P. brasiliensis* GAPDH binds to fibronectin, laminin, and type I collagen [68]. In *Sporothrix schenckii,* a chaperone, GroEL/Hsp60, binds to the extracellular matrix proteins laminin, elastin, fibrinogen, and fibronectin [70]. Satala and coworkers recently added phosphoglycerate mutase to the list of *C. albicans* proteins interacting with host ECM proteins vitronectin and fibronectin [61].

Crowe and coworkers studied *C. albicans* proteins that bind to human plasminogen and identified alcohol dehydrogenase, thiol-specific antioxidant protein, translation elongation, peroxisomal catalase, fructose bisphosphate aldolase, phosphoglycerate kinase, and glyceraldehyde 3-phosphate dehydrogenase [50]. Enolase was also found to bind to plasminogen by another group [52]. Ef-Tu elongation factor and glycerol 3-phosphate dehydrogenase bind to factor H as well as to plasminogen [51,56]. Stie and coworkers identified several proteins in *Cryptococcus neoformans* that aid in invasion by interacting with plasminogen, including subunits of ATP synthase, pyruvate kinase, phosphoglycerate kinase, glucose 6-phosphate isomerase, and nitric oxide dioxygenase [65].

Moonlighting proteins that directly affect the host immune system include elongation factor 2 and superoxide dismutase 3. Both proteins interact with contact system proteins HK, FXII, and prekallikrein (PPK) [48,49]. 6-phosphogluconate dehydrogenase catalyzes the oxidative decarboxylation of 6-phosphogluconate to ribulose 5-phosphate with concomitant reduction of NADP to NADPH in the pentose phosphate pathway and has also been found to bind to components of the contact system [47,48,49]. The high affinity glucose transporter 1 (Hgt1p), a transmembrane sugar transporter, binds complement regulator FH and C4BP, and protects cells from the host’s complement cascade by reduced formation of the potentially lytic terminal complex (TCC) [58].

Like the many enzymes and chaperones that have been found to bind RNA through proteomics studies, many other intracellular proteins have also been found on the cell surface through surface proteomics studies [91]. Many of these proteins might also have moonlighting functions on the cell surface.

## 3. Discussion

The fungal moonlighting proteins described above include a large variety of proteins and combinations of functions. Some combinations of functions enable cells to sense and respond to changes in the environment; for example, proteins involved in redox reactions might sense changes in the redox state of the cell and then switch to a new function. Proteins involved in carbohydrate or nitrogen metabolism are often important sensors for the cellular levels of these nutrients. In general, moonlighting proteins can also enable coordinated regulation or crosstalk between biochemical pathways and cellular processes, such as energy producing pathways and transcription or translation. In other cases, the two functions might not be connected but might just be a case of a new function arising and the cell making use of proteins that are already available.

While we cannot always determine which function was the original function of the protein family, many moonlighting proteins belong to ancient protein families, notably enzymes in glycolysis, the citric acid cycle, and protein synthesis, including ribosomal proteins. Evolution of a new function does not always require large changes in the amino acid sequence or protein structure [92]. In fact, a binding site for plasminogen can be just a few amino acid changes in a protein surface loop or C-terminus. Many of these proteins have orthologues that are moonlighting in other organisms [2]. Interestingly, in some cases the more ancient, catalytic function is the same and a second function is different, a kind of “semi-orthologue.”

There are still many questions about moonlighting proteins. In many cases, very little is known about the molecular mechanism for performing one or both functions and how a protein might either switch between functions or perform both functions simultaneously. Even models or three-dimensional structures of a protein in one conformation or one complex often do not provide enough information about how it performs all of its functions. Structures of proteins in different conformations and in complex with other proteins, DNA, RNA or other ligands or cofactors are needed. In addition, for most of the intracellular proteins that have a second function on the cell surface, it is not known how the proteins can be targeted to these two different cellular locations, although several noncanonical secretion pathways have been found to contribute to intracellular proteins being found on the cell surface (reviewed in [93]).

For some proteins, it is clear from mutagenic studies that a protein has a function in addition to its catalytic function, but the biochemical or biophysical function is not known. For example, the DeLuna lab showed that mutant forms of the *S. cerevisiae* Alt1 and Bat2 transaminases and the isoleucine/valine biosynthetic enzymes Ilv1 and Ilv2 that lack catalytic function can partially restore the gene knockout phenotype [94]. Detomasi and coworkers similarly showed that *N. crassa* CWR-1 is a C1 oxidizing chitin polysaccharide monooxygenase as well as having a function in allorecognition during formation of an interconnected mycelial network. Mutant enzymes that lack the catalytic function still function in allorecognition, likely through protein–protein interactions with CWR-2 [95]. Flores and coworkers showed that deletion of *Yarrowia lipolytica* N-acetylglucosamine kinase (Nag5) results not only in loss of kinase activity but also in altered levels of expression of enzymes of the N-acetylglucosamine (NAGA) utilization pathway (NAG genes). Complementation with mutant Nag5 proteins with greatly decreased kinase activity could restore the regulation of gene expression [96]. These results indicate that Nag5 has separate kinase and gene regulation functions, although how it regulates gene expression, for example, by binding to DNA or to a transcription factor, is not known.

The effects of interactions with additional macromolecules are often not clear. Many enzymes in intermediary metabolism in yeast and other organisms have been found to bind RNA, but in most cases, it is not clear if the interaction affects aspects of RNA function—translation, splicing, localization, or lifetime—or, perhaps, aspects of the enzyme function—catalysis, localization, or degradation.

With such variety of moonlighting proteins and functions, there are not shared characteristics that have been identified for predicting if a protein has moonlighting functions, although some characteristics and methods can be used to accurately predict individual functions. In general, many moonlighting proteins have similar characteristics to other proteins found in the same cellular locations [97]. Many of the new functions were identified by serendipity, but more recently, proteomics studies have identified many examples [98]. For example, proteomics studies that identified proteins that bind to plasminogen or to RNA have identified many of the proteins mentioned above as well as similar moonlighting proteins in other species.

The importance of fungi in health and disease, agriculture, and biotechnology has played a role in the increasing number of moonlighting functions that have been identified in recent years, and studies of the moonlighting functions might be beneficial in these fields. Several of the species mentioned above are important components of the human microbiome, especially the gut microbiome, sometimes as commensal species and sometimes as pathogens. Some of the moonlighting proteins mentioned above are also involved in interfering with therapeutics or treatments, including adhesion to medical implants [99]. Understanding the functions of moonlighting proteins that interact with host proteins and act as adhesins, invasins, or immune system modulators could lead to improved therapeutics or preventative measures. Similarly, therapeutics or preventative measures might be found for treatment of fungal infections of agricultural animals or plants. In contrast, some fungi are important as food or in the production of food molecules, so understanding moonlighting proteins that affect cellular processes such as translation and protein production could be used in improving yields. Moonlighting proteins that sense small molecules and affect translation or transcription could be important starting points for synthetic biotechnology, for example, as sensors that affect translation or transcription.

## 4. Conclusions

Moonlighting proteins in fungi have a variety of functions and combinations of functions. In recent years, the use of proteomics methods to identify cell surface localized proteins or proteins that bind to DNA or RNA has contributed to the identification of dozens of additional moonlighting, or potentially moonlighting, proteins. The examples described above are likely to be only the tip of the iceberg, with additional functions of many more proteins still to be identified. There is still much to learn about the mechanisms of their functions and their cellular roles, with potential impacts in medicine, agriculture, and synthetic biology.

## Figures and Tables

**Table 1:** Moonlighting Proteins Mentioned in the Text.

Protein Name	One Function	Another Function	Reference Number
**Interacting with DNA or affecting DNA metabolism**			
***Emericella nidulans***			
I-AniI	mRNA maturase	Homing endonuclease	[3,4]
***Kluyveromyces lactis***			
Galactokinase	Galactokinase	Transcriptional activator	[5,6]
***Tuber melanosporum***			
Ask1	Component of	Binds DNA,	[7]
	DASH complex	transcriptional regulator	
Phosphoadenosine	Thioredoxin	Transcription factor	[8]
phosphosulfate reductase			
***Saccharomyces cerevisiae***			
Acetohydroxyacid	Acetohydroxyacid	Maintains mitochondrial	[9]
reductoisomerase	reductoisomerase	DNA stability	
Arg5,6	N-acetyl-gamma	Regulator of transcription	[10]
	phosphate reductase/acetyl glutamate kinase		
Arg82	Kinase	Stabilizes transcription factors	[11]
		ARG80 and MCM1	
Glycerol 3-phosphate	Glycerol 3-phosphate	Binds to Sir2	[12]
Dehydrogenase	dehydrogenase		
Hexokinase	Hexokinase	Binds Mig1	[13,14]
Hsp60	Chaperonin	Binds DNA	[15]
Lys20	Homocitrate synthase	DNA damage repair	[16]
Superoxide dismutase	Superoxide dismutase	Binds DNA	[17,18]
Ure2	functions in	Glutathione peroxidase, enzyme	[19,20,21,22]
	nitrogen catabolite repression		
Zuotin	Component of the	Activator of a transcription factor	[23]
	RAC chaperone complex		
**Binding to RNA**			
***Neurospora crassa***			
Tyrosyl tRNA synthetase	Catalyzes aminoacylation	Promotes folding of introns	[24]
	of tyrosine		
***Saccharomyces cerevisiae***			
Isocitrate dehydrogenase 1	Isocitrate dehydrogenase	Binds mRNA	[25]
Isocitrate dehydrogenase 2	Isocitrate dehydrogenase	Binds mRNA	[25]
L2 Ribosomal protein	Component of ribosome	Shortens half-life of L2 mRNA	[26]
L32 Ribosomal protein	Component of ribosome	Inhibits the splicing of RNA	[27]
Leucyl-tRNA synthetase	Leucyl-tRNA synthetase	Intron splicing	[28]
S14 Ribosomal protein	Component of ribosome	Binds RNA, translation repressor	[29]
S28 Ribosomal protein	Component of ribosome	Binds, shortens half-life of mRNA	[30]
**Protein–Protein Interactions**			
***Hansenula poymorpha***			
Pyruvate carboxylase	Pyruvate carboxylase	Assembly of peroxisomal	[31,32]
		alcohol oxidase	
***Pichia pastoris***			
Phosphofructokinase	Phosphofructokinase	Microautophagy	[33]
***Saccharomyces cerevisiae***			
Arginase	Arginase	Binds and inactivates	[34]
		ornithine transcarbamylase	
Cytochrome C	Electron carrier protein	Binds apoptosis proteins	[35]
	in electron transport chain		
Enolase 1	Enolase	Vacuole-membrane fusion	[36]
		and protein trafficking to the vacuole	
Enolase 2	Enolase	Vacuole-membrane fusion	[36]
		and protein trafficking to the vacuole	
Fructose 1,6-bisphosphate	Aldolase	Binds vacuolar H+-ATPase	[37]
aldolase		and is needed for its assembly	
Hal3	Subunit of	Inhibitory subunit of	[38,39]
	phosphopantothenoyl	protein phosphatase PPZ1	
	cysteine decarboxylase		
	coenzyme A		
Peroxiredoxin 1	Peroxidase	Molecular chaperone	[40]
Peroxiredoxin 2	Peroxidase	Chaperone	[40]
Rack1	Component of ribosome	Scaffold in	[41,42]
		signal transduction pathways	
Succinate dehydrogenase	Electron transport in	Component of TIM22 complex	[43,44,45,46]
subunit 3	respiratory complex II		
**Cell Surface**			
***Candida albicans***			
6-phosphogluconate	Oxidative decarboxylation	Binds components of contact system	[47,48,49]
dehydrogenase	of 6-phosphogluconate		
Alcohol dehydrogenase 1	Alcohol dehydrogenase	Binds plasminogen	[50]
Ef-Tu	Elongation factor	Binds plasminogen,	[48,49,51]
	during protein synthesis	factor H, HK, FXII, and prekallikrein	
Enolase	Enolase, enzyme	Binds plasminogen	[52]
Fructose bisphosphate	Fructose bisphosphate	Binds plasminogen	[50]
aldolase	aldolase		
Glyceraldehyde	Glyceraldehyde	Binds plasminogen,	[53]
3-phosphate	3-phosphate	fibronectin, and laminin	
dehydrogenase	dehydrogenase		
Glutathione reductase	Glutathione reductase	Binds plasma proteins	[54,55]
Glycerol 3-phosphate	Glycerol 3-phosphate	Binds plasminogen	[56]
dehydrogenase	dehydrogenase		
Heat shock protein Ssa2	Chaperone	Binds a peptide in saliva	[57]
High-affinity glucose	Glucose transporter	Complement inhibitor	[58]
transporter 1			
Integrin-like protein	Inhibits opsonization	Adhesin	[59,60]
	and phagocytosis		
Peroxisomal catalase	Peroxisomal catalase	Plasminogen binding	[50]
Phosphoglycerate kinase	Phosphoglycerate kinase	Plasminogen binding	[50]
Phosphoglyceromutase	Phosphoglyceromutase	Binds plasminogen, vitronectin, and fibronectin	[50,61]
Phosphotransferase	Transports and	Binds to plasma proteins	[48,62]
	phosphorylates sugars		
Superoxide dismutase 3	Removes superoxide	Binds contact system proteins	[48,49]
	radicals	HK, FXII, and prekallikrein	
Thiol-specific antioxidant	Thiol-specific antioxidant	Binds plasminogen	[50]
protein	protein		
Transcription elongation	Transcription elongation	Binds plasminogen	[50]
factor	factor		
Triosephosphate	Isomerase	Binds extracellular matrix proteins	[61]
isomerase			
Heat shock protein Ssa2	Chaperone	Binds HTN3/histatin 5	[57]
***Candida glabrata***			
Triosephosphate	Isomerase	Binds extracellular matrix proteins	[61]
isomerase			
***Candida tropicalis***			
Enolase	Enolase	Binds vitronectin, fibronectin,	[63,64]
		and plasminogen	
Fructose-bisphosphate	Aldolase	Binds fibronectin,	[64]
aldolase		vitronectin, and laminin	
***Cryptococcus neoformans***			
Glucose 6-phosphate	Glucose 6-phosphate	Binds plasminogen	[65]
isomerase	isomerase		
Nitric oxide dioxygenase	Nitric oxide dioxygenase	Binds plasminogen	[65]
Phosphoglycerate kinase	Phosphoglycerate kinase	Binds plasminogen	[65]
Pyruvate kinase	Pyruvate kinase	Binds plasminogen	[65]
Subunits of ATP synthase	ATP synthesis	Binds plasminogen	[65]
***Histoplasma capsulatum***			
Hsp60 chaperone	Chaperone	Adhesin	[66]
***Paracoccidioides brasiliensis***			
Enolase	Enolase	Binds fibronectin	[67]
Glyceraldehyde	Glycerol 3-phosphate	Binds fibronectin, laminin,	[68]
3-phosphate	dehydrogenase	and type I collagen	
dehydrogenase			
Triose phosphate	Triose phosphate	Adhesin	[69]
isomerase	isomerase		
*Sporothrix schenckii*			
GroEL/Hsp60	Chaperone	Binds laminin, elastin,	[70]
		fibrinogen, and fibronectin	
***Talaromyces marneffei***			
Glyceraldehyde	Glyceraldehyde	Adhesin	[71]
3-phosphate	3-phosphate		
dehydrogenase	dehydrogenase		

## Data Availability

No new data were created or analyzed in this study.
